# Enduring Benefits of Widespread TMS Implementation: Analysis of Data in Pennine Care NHS Foundation

**DOI:** 10.1192/bjo.2024.470

**Published:** 2024-08-01

**Authors:** Donia El-Nemr, Stephanie Murch, Micheal Kurkar, Andi Stanescu, Syed Haque

**Affiliations:** Pennine Care NHS Foundation Trust, Greater Manchester, United Kingdom

## Abstract

**Aims:**

Transcranial Magnetic Stimulation (TMS), characterized by its non-invasiveness and absence of recovery time, emerges as an optimal intervention for treatment-resistant depression. Operating through the induction of a time-varying electric field in the brain, TMS elicits action potentials in cortical neurons, leading to long-term neural inhibition and excitation, fostering neuroplasticity. Despite its efficacy, TMS remains available in a limited number of National Health Service (NHS) hospitals. This study aims to evaluate the use of TMS for treatment resistant depression and its impact upon service utilisation within Pennine Care NHS Foundation Trust.

**Methods:**

A retrospective analysis was conducted on 76 patients diagnosed with treatment-resistant depression. Responders (n = 54) and non-responders (n = 22) were identified based on baseline, midpoint and endpoint assessments using HDRS, Beck's Inventory, PHQ-9, and GAD anxiety questionnaires. Patient data was extracted from PARIS, the Electronic Patient Record system of Pennine Care NHS Foundation, encompassing NHS service utilisation pre- and post-TMS treatment.

**Results:**

Comparison between 12 months pre and post-TMS treatment revealed noteworthy findings:

12 responders (22%) were admitted to hospital in the year prior to starting treatment with a total of 1134 and mean of 94.5 days. In comparison to post-TMS where 11 (20.4%) patients had total of 913 and mean of 83 days.

8 non-responders (36.4%) were admitted to hospital in the year prior to starting treatment with a total of 285 and mean of 36.5 days. In comparison to post-TMS where 3 (13.6%) patients had a total of 276 and mean of 92 days.

Outpatient appointments reduced by 15.4% for responders and 27.2% for non-responders.

Number of A&E admissions reduced by 79.3% for responders and 65.5% for non-responders.

Admissions to Home Treatment Team (HTT) decreased by 62.7% for responders and 86.7% for non-responders.

Post-TMS discharge from services was 25.9% for responders and 18.2% for non-responders.

**Conclusion:**

This study underscores a reduction in service utilisation among treatment-resistant depression patients following TMS treatment, with some indication that a greater reduction is seen for responders to treatment. While there was limited benefit seen when analysing outpatient appointments and HTT involvement, a greater reduction was seen when evaluating A&E attendance and days spent in hospital. In addition to exploring the possibility of late response to treatment and how this affects non-responder data, future studies are needed to compare results with patients who did not have TMS. These studies will require larger study numbers to better analyse the enduring benefits of widespread TMS implementation within the NHS.

